# Impact of Human Granulocytic Anaplasmosis in Spain from 1997 to 2022

**DOI:** 10.3390/tropicalmed10070183

**Published:** 2025-06-29

**Authors:** Hugo Almeida, Montserrat Alonso-Sardón, Beatriz Rodríguez-Alonso, Amparo López-Bernus, Ángela Romero-Alegría, Virginia Velasco-Tirado, Antonio Muro, Moncef Belhassen-García

**Affiliations:** 1 Servicio de Medicina Interna, Hospital Universitario de Salamanca (HUS), Centro de Investigación de Enfermedades Tropicales de la Universidad de Salamanca (CIETUS), Instituto de Investigación Biomédica de Salamanca (IBSAL), Universidad de Salamanca, 37007 Salamanca, Spain; hugoalmeida6@gmail.com; 2 Área de Medicina Preventiva, Epidemiología y Salud Pública, Facultad de Medicina, Centro de Investigación de Enfermedades Tropicales de la Universidad de Salamanca, Instituto de Investigación Biomédica de Salamanca, 37007 Salamanca, Spain; sardonm@usal.es; 3 Servicio de Medicina Interna, Hospital Universitario de Salamanca, Centro de Investigación de Enfermedades Tropicales de la Universidad de Salamanca, Instituto de Investigación Biomédica de Salamanca, 37007 Salamanca, Spain; aralegria@yahoo.es; 4 Servicio de Medicina Interna, Unidad de Enfermedades Infecciosas, HUS, CIETUS, IBSAL, 37007 Salamanca, Spain; alopezb@saludcastillayleon.es; 5 Servicio de Dermatología, Hospital Universitario de Salamanca, Centro de Investigación de Enfermedades Tropicales de la Universidad de Salamanca, Instituto de Investigación Biomédica de Salamanca, 37007 Salamanca, Spain; virvela@yahoo.es; 6 Grupo de Enfermedades Infecciosas y Tropicales (e-INTRO), Centro de Investigación de Enfermedades Tropicales de la Universidad de Salamanca, Instituto de Investigación Biomédica de Salamanca, Facultad de Farmacia, Universidad de Salamanca, 37007 Salamanca, Spain; ama@usal.es; 7 Servicio de Medicina Interna, Unidad de Infecciosas, Hospital Universitario de Salamanca, Centro de Investigación de Enfermedades Tropicales de la Universidad de Salamanca, Instituto de Investigación Biomédica de Salamanca, Universidad de Salamanca, 37007 Salamanca, Spain

**Keywords:** human granulocytic anaplasmosis, tick-borne diseases, zoonosis, epidemiology, Spain

## Abstract

Human granulocytic anaplasmosis (HGA) is an emerging zoonosis in Europe, with limited data available from Spain. This study aimed to quantify HGA cases in the Spanish National Health System over the last 26 years, assess its evolution, and evaluate the impact in terms of hospital stay and cost. A retrospective observational case series was conducted using the Minimal Basic Data Set (MBDS, CMBD in Spanish). Hospitalized patients with ICD-9-CM and ICD-10 codes for anaplasmosis from 1997 to 2022 were included. Ten HGA cases were reported. The incidence remained stable at one case per year [IR = 0.021 cases/million person-years], except in 2018 [IR = 0.048]. Six patients (60%) were men, with a mean age of 49 years (±20.9). Hospital stays ranged from 3 to 13 days. The total cost was EUR 45,540.57, with an average cost of EUR 4554.06 (±1032.16) per case. All patients had favorable outcomes. HGA has a low incidence in Spain, with moderate associated costs. Despite its emergence, its economic and health impact remains manageable, underscoring the effectiveness of Spain’s healthcare system. Continued surveillance and prevention are essential to address evolving vector-borne diseases.

## 1. Introduction

Anaplasmosis, also known as human granulocytic anaplasmosis (HGA), is a tickborne disease caused by the bacterium *Anaplasma phagocytophilum*. *Anaplasma* species are obligate intracellular *rickettsial* pathogens that cause significant diseases in animals and humans such as acute, nonspecific febrile illness [1]. The genus *Anaplasma* currently comprises at least eight recognized species. Although *A. phagocytophilum* is the primary agent of HGA, sporadic zoonotic infections have also been reported for *A. capra* and *A. ovis* [2,3]. Within *A. phagocytophilum* itself, multilocus sequence typing reveals distinct ecotypes differing in host range and pathogenicity: the Ap-ha variant predominates in North American human cases, whereas European and Asian strains form several clonal complexes, only some of which infect humans [4,5]. European studies show that strains infecting humans tend to cluster separately from those found in wildlife such as roe deer and small mammals [6]. Although the overall genetic diversity of *A. phagocytophilum* appears to be greater in Europe than in the United States, the strains responsible for human infections in both regions are phylogenetically similar, suggesting limited variation in pathogenicity among the human-infective subset [7].

HGA is an emerging zoonosis transmitted in our environment through the bite of *Ixodes ricinus* (Ixodidae tick). Ticks are haematophagous parasites with a global distribution and play crucial roles from an epidemiological and clinical standpoint, given their propensity to transmit a wide variety of pathogens including viruses, bacteria, and protozoa to both animals and humans. In the context of Spain, particularly in the northwest, the increasing prevalence and diversity of tick species, along with observed climate changes, predict an increase in the incidence of diseases transmitted by these arthropods [8,9,10]. Despite the increasing number of studies on *A. phagocytophilum* genetic diversity, there are still insufficient data to understand the geographical distribution, host preferences, and pathogenicity of each described genetic variant [7].

In Europe, *A. phagocytophilum* is widely present in ticks and various animal hosts, both wild and domestic. Despite this, HGA remains rare in Europe, with fewer than 300 clinical cases reported [1,7]. In Spain, only a limited number of cases have been reported in the literature [7,11,12]. This is in stark contrast to the United States, where over 15,000 cases were documented by 2015, following a steady rise since 2001 [13]. This disparity raises concerns about whether the actual incidence of HGA in Europe is accurately reflected, or if the disease is underdiagnosed or underreported. Epidemiological studies indicate that certain groups, such as forestry workers, hunters, veterinarians, and farmers, especially those with a history of tick bites or those who live in endemic areas, are at increased risk of infection [14,15,16,17]. Co-infection with other pathogens transmitted by *I. ricinus*, particularly *Borrelia burgdorferi*, has also been identified as a potential risk factor for HGA [13,14,18].

In Europe, HGA typically presents as mild infections with symptoms such as fever, headache, myalgia, and arthralgia [7]. The comparison between the low number of reported cases and the seroprevalence data in humans, which ranges between 8.3% and 31%, is significant [19,20,21,22]. Serological surveys show markedly higher exposure: pooled European estimates reach 8% in the general population, with local figures of 7.5% in forestry workers from Bavaria, Germany, 4–8% in residents of Berlin–Brandenburg, Germany, and similar values reported in Sweden and Italy [19,20,21,22,23]. The discrepancy between these seroprevalence data and the few published clinical cases strongly suggests under-diagnosis or underreporting of HGA in Europe [7].

In this context, it is difficult to analyze the relevance of these genetic groups for public health. Moreover, despite several recent reviews, epidemiological data regarding human infections in Europe are poorly collated, consisting of a collection of case reports and seroprevalence studies. Despite their importance, limited information on *Anaplasma* spp. infections in Spain has been published thus far [24,25,26].

This study represents the first epidemiological investigation of HGA in Spain. The aim of this study was to quantify the number of cases of HGA registered in the Spanish National Health System over the last twenty-five years, to describe its evolution over time, and to assess its impact in terms of average length of hospital stay and treatment cost.

## 2. Materials and Methods

### 2.1. Study Design, Setting, Case Selection and Data Collection

A retrospective, observational case series was designed to identify HGA in Spanish National Health System hospitals from 1 January 1997 to 31 December 2022. Data were obtained from the Minimal Basic Data Set (MBDS) or *Conjunto Mínimo Básico de Datos* (CMBD in Spanish), a specialized health registry that uses each patient care contact as a recording unit and is an essential resource for epidemiological research. It is a clinical-administrative registry based on discharge reports, mandatory for all public hospitals included in the Spanish National Health System. Private hospitals are only included if they have agreements with the National Health System. Primary healthcare centers and general practitioners do not contribute directly to this registry, as their diagnoses are recorded in separate outpatient systems.

CMBD includes variables that identify the care provider (center, unit, physician), the patient (medical record number, health card number, etc.), the diagnostic and therapeutic procedures performed during hospital care, the length of stay and the average cost of the hospitalization process; it does not collect clinical signs, laboratory values or medication data [27].

From its creation in 1997 until 2015, the CMBD applied the Clinical Modification of the 9th Revision of the International Classification of Diseases (ICD-9-CM) as the reference classification for coding diagnoses and procedures. Since 1 January 2016, the RAE-CMBD has adopted the ICD-10 as the reference classification for the clinical coding and registration of morbidity cases treated in the Spanish National Health System. The tabular list of the International Classification of Diseases has categorized HGA under the subheading “tick-borne rickettsiosis/other ehrlichiosis,” ICD-9 code 082.49 (from 1997- to 2015), and ICD-10 code A77.49 (from 2016- to 2022). Because the Spanish ICD-10 (CIE10-ES) does not include the North American sub-code A79.82, admissions were identified with the only *Anaplasma-specific* code available, A77.49 “Other rickettsioses due to *Anaplasma phagocytophilum*”, and, for 1997–2015, the analogous ICD-9 codes 082.40/082.41. Records were retained only when (i) microbiological confirmation of *A. phagocytophilum* by PCR or serology was coded and (ii) no *Ehrlichia* code appeared in any diagnostic field (*Ministerio de Sanidad*, 2022).

Principal diagnosis is defined as the condition after the study that caused the admission to the hospital, according to the ICD-9-CM or ICD-10 Official Guidelines for Coding and Reporting and according to the criteria of the attending clinical service or medical practitioner, even if major complications or other independent conditions have occurred during the stay. Secondary diagnoses are “other diagnoses” or conditions that coexist with the principal condition at the time of admission (i.e., comorbidities) or develop later during the hospital stay (i.e., complications), thus influencing the length of stay or the treatment administered. Both were included in this report.

CMBD also provides the estimate of the average costs of hospitalization processes according to the All-Patient Diagnosis Related Group (AP-DRG) classification system. The estimated data for each patient include hospital stay, medical investigations (radiology, laboratory tests) and specific treatments related to the main diagnosis, grouped together (not broken down/disaggregated or specified). However, indirect costs, such as lost income or sick leave, were not included.

Population estimates were obtained from the Spanish National Statistics Institute (INE), using annual population figures for the period 1997–2022. These data provide official population counts for Spain, ranging from 39,323,320 inhabitants in 1997 to 47,475,420 in 2022 [28].

### 2.2. Data Analysis

Statistical analysis was performed with IBM SPSS Statistics 28.0 and figures were constructed using Jamovi statistical software (Version 2.0). We first conducted a descriptive analysis of the variables. Counts and proportions were used to define the qualitative variables. The means with standard deviations (SDs) and median values and the 25th and 75th percentile values (interquartile ranges; IQRs) were used to show the results of the quantitative variables. Qualitative variables were tested with Pearson’s chi-square test to assess trends. ANOVA was applied to determine the existence of statistically significant mean differences between two or more categorical groups. The Pearson correlation coefficient (r) was used to describe the strength and direction of the linear relationship between two quantitative variables. Statistically significant differences were those where *p* <  0.05. The incidence rate (IR) was calculated to express the number of cases per person per year of examination (cases per million person-years).

## 3. Results

### 3.1. Epidemiological Features of the Case Series

Throughout these 26 years of study, a total of 10 infection episodes have been reported in the Spanish National Health System. Four cases (40%) were the principal diagnoses, five (50%) were secondary diagnoses, and one episode (10%) was a reactivation/recurrence of a previous case (two months later), so the sample was composed of nine patients. Of these, six (66.7%) were men and three (33.3%) were women. The study participants’ mean (±SD) age was 53 years (±18.2), and the median age was 63 years [IQR: 66–41], ranging from 15 to 69 years. The main descriptive data of this case series are shown in Table 1. Two episodes recorded in 2018 corresponded to the same patient, a 15-year-old female from Castilla y León, who was admitted in August due to Q fever as primary diagnosis and anaplasmosis as secondary diagnosis and readmitted in October for reactivation/recurrence of symptoms.

### 3.2. Clinical Features of the Infection Episodes

**Figure 1:**
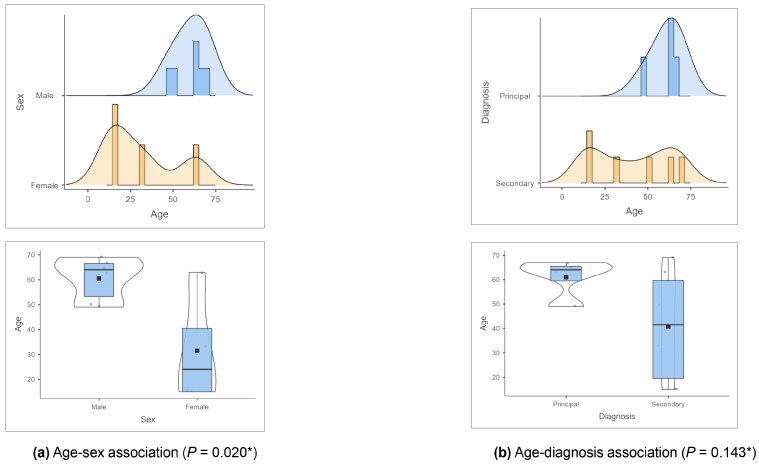
Age distribution and relationship with other variables: (**a**) age–sex association, (**b**) age–diagnosis association. (* ANOVA).

When the 10 infection episodes were analyzed, significant differences (*p* = 0.02) were observed in the mean ages of men and women: 60.5 (±8.7) and 31.5 (±22.6), respectively. The principal diagnosis was also more common in older patients, though it was not statistically significant (*p* = 0.143); the mean age at principal diagnosis [61 (±8.1) years] was compared to that at secondary diagnosis [41 (±23.5) years] (Figure 1).

**Figure 2:**
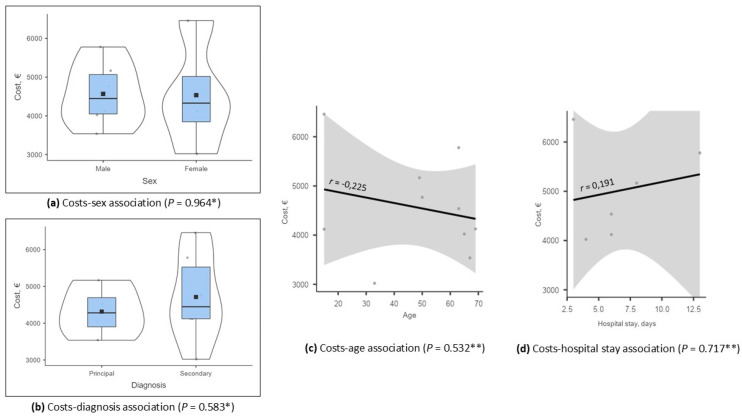
Association of healthcare costs with other variables: (**a**) costs–sex association, (**b**) costs–diagnosis association, (**c**) costs–age association, (**d**) costs–hospital stay association (* ANOVA. ** Pearson correlation coefficient).

Of all hospitalized patients, 8 were urgently hospitalized and 2 were scheduled, most often in the internal medicine department/service (8). Hospital stays ranged from 3 to 13 days, with a mean of 7 days (±3.5). The total cost of anaplasmosis in Spain was EUR 45,540.57, with a mean cost per case of EUR 4554.06 (±1032.16). No significant differences were observed in terms of health costs [average cost for men was EUR 4567.07 (±826.54) compared to EUR 4534.53 (±1434.09) for women (*p* = 0.964)]. Similarly, the average cost for the principal diagnosis was 4316.43 € (±699.25) compared to 4712.47 € (±1244.92) for the secondary diagnosis (*p* = 0.583). The Pearson correlation coefficient revealed a negative correlation between age and hospital costs: the lower the age was, the higher the hospital costs (r = −0.225; *p* = 0.532). A weak positive correlation between average hospital stay and hospital costs was observed; hospital costs increased with the duration of hospital stay (r = 0.191; *p* = 0.717) (Figure 2). All patients had a favorable outcome (0% mortality).

### 3.3. Incidence Data

The overall reported incidence rate (1997–2022) was 0.008 cases per million person-years. The first hospital registry was created in 2008 (IR = 0.022 cases per million person-years). From this date, the incidence of cases was homogeneous throughout the study period, with one record per year [IR = 0.021 cases per million person-years].

The Spanish region with the highest incidence of cases was Castilla y León, where 2 cases were reported (IR = 0.031 cases per million person-years). The incidence rates in the other regions were as follows: Aragón (IR = 0.030 cases per million person-years), Murcia (IR = 0.028 cases per million person-years), Galicia (IR = 0.014 cases per million person-years), Madrid (IR = 0.013 cases per million person-years), Andalucía (IR = 0.005 cases per million person-years), and Cataluña (IR = 0.005 cases per million person-years). All the patients presented various comorbidities, such as other zoonoses: Q fever, babesiosis, leptospirosis and Lyme disease. Only one patient was diagnosed with anaplasmosis as the only diagnosis (i.e., without comorbidities). Detailed data for each of the cases is shown in Table 2.

## 4. Discussion

In Spain, *Anaplasma phagocytophilum* circulates in a complex enzootic cycle dominated by the tick *Ixodes ricinus*. Infection prevalence in questing ticks ranges from 0.5% in Mediterranean scrublands to 15% in humid Atlantic forests [8]. Roe deer, red deer and wild boar act as major reservoirs, with PCR or seropositivity rates of 8–35%, while domestic sheep and cattle show lower but consistent exposure (4–12%) [11]. Larval and nymphal stages feed on small mammals and birds, sustaining trans-stadial transmission, whereas adults favor large ungulates, amplifying tick density; humans and dogs are incidental hosts, usually infected by nymphs during spring–summer [29].

Although research on the genetic diversity of *A. phagocytophilum* has increased, the current data remain insufficient to fully elucidate the geographical distribution, host preferences, and pathogenicity of the various genetic variants identified. This lack of comprehensive data hampers our ability to understand the epidemiology and potential public health implications of each variant [7].

Our study identified a total of 10 admissions over a 26-year interval, underscoring the emerging nature of HGA in Spain and its substantial impact in terms of hospital stay and associated costs, with an average cost of EUR 4554.06 per case. This low incidence and moderate cost may differ from data reported in other countries. For example, in the United States, the incidence of HGA was 6.3 cases per million person-years between 2008 and 2012, with states such as Minnesota and Wisconsin reporting much higher rates [16]. Given that a higher number of identified cases is expected to be associated with increased overall costs, direct comparisons require consideration of both incidence rates and cost per case, impossible with this study. Additionally, the reported hospitalization rate in the U.S. was 31%, indicating greater severity.

In other parts of the world, such as Korea, 17 patients were identified between 2015 and 2018, with incidence peaks in May and June, suggesting seasonality and a possible increase in transmission during certain times of the year [30]. These studies underscore the regional variability in the prevalence and impact of HGA, highlighting the importance of regionally adapted prevention and diagnostic strategies [31]. The variability in incidence patterns and associated costs reflects differences in vector exposure, diagnostic practices, and possibly disease surveillance. The absence of vaccines for HGA highlights the potential cost-effectiveness of preventive measures in individuals at risk of tick bites, particularly in autonomous communities with higher incidences of HGA, such as Madrid and Castilla y León.

Co-infection is increasingly recognized as the rule rather than the exception in *Ixodes*-endemic areas. Some series report concomitant pathogens in 40–70% of HGA cases, most commonly *Borrelia burgdorferi* sensu lato, *Babesia divergens* and, less often, *Coxiella burnetii* [7,19]. European reviews place the proportion of laboratory-confirmed co-infection at a similar 30–50% [25]. In our cohort, no cases of co-infection with other tick-borne pathogens were identified, underscoring the ecological overlap among these agents and reinforcing the rationale for multiplex diagnostic strategies, even when co-infections are not observed. The geographical distribution of our nine patients mirrors the animal reservoir: serosurveys reveal the highest *A. phagocytophilum* exposure (>10%) in livestock, dogs, and wild deer from the Atlantic and north–central regions of Spain—Galicia, Castilla y León, Cantabria, and the north of Madrid [11,32]. Seven of the nine human cases originated in those same autonomous communities, supporting a shared eco-epidemiological focus.

The presence of *A. phagocytophilum* in Europe is significant [7,33], although clinical cases of HGA are rare. This discrepancy may be due to under-diagnosis or underreporting. The genetic diversity of *A. phagocytophilum* is greater in Europe than in the USA, but the strains that infect humans are related in both regions. In Europe, the diagnosis of HGA often requires laboratory confirmation before treatment to avoid antibiotic overuse, unlike in the USA, where to avoid complications associated with some tickborne rickettsial diseases, treatment is often given before the results of laboratory tests are available [7,25]. This difference in diagnostic and treatment practices underscores the need for regionally adapted approaches for managing HGA.

The increased desire for outdoor recreational activities has increased the exposure to potential human pathogens that previously cycled almost exclusively within natural, nonhuman enzootic hosts [34,35].

The incidence rate observed in our study suggests that HGA, while not widespread, poses a consistent health challenge in Spain. This aligns with findings from Dumic et al., who noted the rise in HGA in Europe, indicating an increased need for awareness and diagnostic readiness among healthcare professionals [25]. While HGA incidence was stably low, this pattern still fits within the wider increase in vector-borne zoonoses—such as Lyme borreliosis and Crimean-Congo haemorrhagic fever—documented in Spain over the same period [7,32,36,37,38,39]. The alteration in natural habitats and changing patterns of human mobility increase human contact with tick vectors. Additionally, global warming might facilitate the geographic expansion of ticks into areas previously less hospitable, thus broadening the range of pathogen transmission such as *A. phagocytophilum* [8,26]. These changes underscore the need to strengthen epidemiological surveillance and develop proactive strategies to mitigate the impact of these vector-borne diseases. Preventive and educational measures aimed at the most exposed communities are crucial to reduce the incidence of bites and improve the early detection of tick-borne diseases.

## 5. Conclusions

This nationwide, hospital-based analysis demonstrates that human granulocytic anaplasmosis remains an exceedingly rare cause of admission in Spain—averaging 0.008 cases per 100,000 population-year between 1997 and 2022—but incurs a median direct cost of EUR 4400 per episode. Although incidence was stable, comparison with seroprevalence data and out-patient figures from other countries suggests under-diagnosis. Sustained epidemiological surveillance and targeted clinical awareness are therefore warranted to ensure timely recognition and management of this emerging tick-borne infection.

### Limitations and Strengths

Despite the small number of cases described, this research contributes valuable insights into the prevalence and characteristics of Anaplasma spp. infections in Spain and emphasizes the need for further research and enhanced surveillance to better understand and manage these diseases.

Because the CMBD records only completed hospital admissions, cases managed exclusively in out-patient settings are not captured, a limitation that probably leads to under-ascertainment of non-severe HGA (compare the 60–70% out-patient management reported in the United States [16]).

In the United States, where all laboratory-confirmed HGA cases are notifiable irrespective of care setting, only 31% of 5762 cases recorded between 2008 and 2019 were hospitalized [16]. Restricting our analysis to CMBD discharge records is therefore likely to capture at most one-third of diagnosed infections and to underestimate the overall incidence.

## Figures and Tables

**Table 1:** Collective summary of the main data of the case series.

1. Epidemiological features of the case series (N = 9)		
Qualitative variables		n (%)
Gender	Male	6 (66.7)
	Female	3 (33.3)
Regions, Autonomous Community	Andalucía	1 (11.1)
	Aragón	1 (11.1)
	Castilla y León	2 (22.2)
	Cataluña	1 (11.1)
	Galicia	1 (11.1)
	Madrid	2 (22.2)
	Murcia	1 (11.1)
**Quantitative variables**	**Mean (± SD), median (IQR), (range, min. value-max. value)**	
Age	53 (± 18.2), 63 (66–41), (range, 15–69)	
**2. Clinical features of the infection episodes (N = 10)**		
**Qualitative variables**		**n (%)**
Type of admission	Urgent	8 (80.0)
	Programmed	2 (20.0)
Service	Internal medicine	8 (80.0)
	Nephrology	1 (10.0)
	Urology	1 (10.0)
Hospital discharge	Home	10 (100.0)
Diagnosis	Principal diagnosis	4 (40.0)
	Secondary diagnosis	5 (50.0)
	Recurrence/reactivation	1 (10.0)
**Quantitative variables**	**Mean (± SD), median (IQR), (range, min. value-max. value)**	
Hospital stays, days	7 (±3.5), 6 (9–4), (range, 3–13)	
Cost, EUR	4554.06 (±1032.16), 4331.83 (5319.61–3902.50), (range, 3020.82–6459.88)	

**Table 2:** Detailed data for each of the cases.

Number of Cases	Year	Month	Gender	Age	Origin	Admission Type	Hospital Service	Diagnosis	Comorbidity	Hospital Discharge	Hospital Stays, Days	Cost (€)
Case 1	2008	July	Male	67	Madrid	Urgent	Internal medicine	Principal	Heart failure, muscular dystrophy, prostate hyperplasia	Home		3538.14
Case 2	2009	January	Male	69	Murcia	Programmed	Urology	Secondary	Bladder neoplasia, hypertension, chronic kidney disease	Home		4126.58
Case 3	2013	May	Female	33	Castilla y Leon	Programmed	Nephrology	Secondary	Connective tissue disease, HTA	Home		3020.82
Case 4	2015	September	Male	50	Madrid	Urgent	Internal medicine	Secondary	Cirrhosis, Ischemic heart disease	Home		4768.43
Case 5	2016	May	Male	65	Galicia	Urgent	Internal medicine	Principal	No comorbidity	Home	4	4023.96
Case 6	2017	August	Male	63	Andalucía	Urgent	Internal medicine	Secondary	Atrial fibrillation, hypertension, pacemaker	Home	13	5778.79
Case 7	2018	August	Female	15	Castilla y Leon	Urgent	Internal medicine	Secondary	Autoimmune lymphoproliferative syndrome	Home	3	6459.88
Case 8	2018	October	Female	15	Castilla y Leon	Urgent	Internal medicine	Secondary	Hepatosplenomegaly	Home	6	4120.34
Case 9	2021	June	Male	49	Aragón	Urgent	Internal medicine	Principal	Purpura	Home	8	5166.56
Case 10	2022	November	Female	63	Cataluña	Urgent	Internal medicine	Principal	Syncope	Home	6	4537.08

## Data Availability

The original contributions presented in this study are included in the article. Further inquiries can be directed to the corresponding authors.
